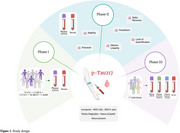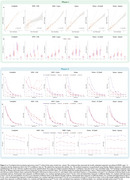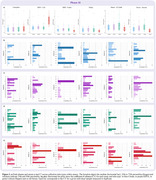# Clinical use of blood ptau217: plasma or serum? The matrix matters

**DOI:** 10.1002/alz70861_108733

**Published:** 2025-12-23

**Authors:** Andrea Benedet, Burak Arslan, Kübra Tan, Hanna Huber, Ilaria Pola, Guglielmo Di Molfetta, Hlin Kvartsberg, Shorena Janelidze, Kaj Blennow, Henrik Zetterberg, Oskar Hansson, Pedro Rosa‐Neto, Nicholas J. Ashton

**Affiliations:** ^1^ McGill University Research Centre for Studies in Aging, Montreal, QC Canada; ^2^ Department of Psychiatry and Neurochemistry, Institute of Neuroscience and Physiology, The Sahlgrenska Academy, University of Gothenburg, Mölndal Sweden; ^3^ Department of Psychiatry and Neurochemistry, Institute of Neuroscience and Physiology, The Sahlgrenska Academy, University of Gothenburg, Mölndal, Gothenburg Sweden; ^4^ Institute of Neuroscienace and Physiology, University of Gothenburg, Mölndal, Västra Götaland Sweden; ^5^ German Center of Neurodegenerative Diseases (DZNE), Bonn, North Rhine‐Westphalia Germany; ^6^ Department of Psychiatry and Neurochemistry, Institute of Neuroscience and Physiology, the Sahlgrenska Academy at the University of Gothenburg, Mölndal Sweden; ^7^ Clinical Memory Research Unit, Department of Clinical Sciences Malmö, Faculty of Medicine, Lund University, Lund Sweden; ^8^ Sorbonne Université, INSERM U1127, CNRS 7225, Paris Brain Institute ‐ PBI, Paris France; ^9^ Department of Neurodegenerative Disease, UCL Institute of Neurology, London UK; ^10^ Clinical Memory Research Unit, Department of Clinical Sciences, Lund University, and Memory Clinic, Skåne University Hospital, Malmö Sweden; ^11^ McGill University Research Centre for Studies in Aging, Douglas Research Centre, Montreal, QC Canada; ^12^ Banner Alzheimer's Institute, Phoenix, AZ USA

## Abstract

**Background:**

Several studies on both research and clinical cohorts have confirmed the high accuracy of plasma *p* ‐Tau217 assays in identifying individuals in the Alzheimer’s disease pathway. However, it is worth noting that while these blood tests rely strictly on EDTA plasma, clinical settings and some bio‐banked research environments often prefer serum matrices, as dictated by established clinical chemistry protocols for other targets. Yet, the performance of *p* ‐Tau217 assays in serum remains unclear, prompting the interest to determine if serum results are comparable to plasma.

**Method:**

This study evaluated the performance of six high‐performing plasma *p* ‐Tau217 assays (Simoa, ALZpath/Janssen; Nulisa, Alamar; Lumipulse, Fujirebio; MSD, Lilly/S‐plex) in three study‐phases (Figure 1). First, *p* ‐Tau217 was quantified and compared between plasma and serum in samples of 100 participants with known amyloid PET status and ranging within the AD continuum. The second phase then assessed specific validation parameters (e.g., Precision, Parallelism, Dilution Linearity, Sample Stability) between matrices within the same assay, following standard immunoassay validation protocols. Phase III compared the impact of different plasma collection tubes (e.g., EDTA, Sodium‐Citrate and Lithium‐Heparin) on *p* ‐Tau217 measurements.

**Result:**

High correlations of ptau217 were found between plasma and serum for most assays, but plasma often yielded higher relative concentrations compared to serum, except for Lumipulse and MSD‐Lilly (Figure 2). In the AD context, ptau217 could detect amyloid pathology with high accuracy in serum (0.63<AUC<0.95). However, plasma cutoffs applied in serum led to high misclassification rates. Furthermore, the reassessment of assay parameters suggest that not all assays can reliably quantify blood ptau217 in serum as they do in plasma. Lastly, ptau217 quantifications highly correlated within assays amongst plasma collection tube types (0.87<rho<0.99), although a proportional bias was detected between plasma EDTA and Citrate (Figure 3).

**Conclusion:**

Our data show that, when assays are reliable in serum, relative biomarker values vary across blood fractions, which most often prevents the use of a single cutoff for the two matrices. In addition, very high agreement was found between collection tube types, suggesting that ptau217 is similarly quantified regardless of the clotting factor used. However, once again the data underscores caution when merging results from samples collected under different protocols.